# Neighborhood Material Deprivation Is Associated with Childhood Asthma Development: Analysis of Prospective Administrative Data

**DOI:** 10.1155/2019/6808206

**Published:** 2019-05-19

**Authors:** Elinor Simons, Sharon D. Dell, Rahim Moineddin, Teresa To

**Affiliations:** ^1^Clinical Epidemiology & Health Care Research, University of Toronto, Toronto, ON, Canada; ^2^Child Health Evaluative Sciences, Hospital for Sick Children, Toronto, ON, Canada; ^3^Department of Family and Community Medicine, University of Toronto, Toronto, ON, Canada; ^4^Dalla Lana School of Public Health, University of Toronto, Toronto, ON, Canada

## Abstract

**Rationale:**

Material deprivation has been proposed as a more comprehensive measure of socioeconomic status than parental income. Stronger associations between childhood emergency department visits for asthma and air pollution have been demonstrated among children living in neighborhoods with high levels of deprivation, but the associations with asthma development and ongoing asthma are not known.

**Objectives:**

We determined the associations between neighborhood material deprivation and the development of new and ongoing childhood asthma.

**Methods:**

Prospectively collected administrative data housed at the Institute for Clinical Evaluative Sciences were examined for Toronto children born from 1997 to 2003. Neighborhood material deprivation, comprising no high school graduation, lone parent families, government transfers, unemployment, low income, and homes needing major repairs, was reported in the Ontario Marginalization Index. Incident asthma was defined by the time of entry into the Ontario Asthma Surveillance Information System (OASIS) database. We measured the risk of incident asthma using Cox proportional hazards models and the associations between ongoing asthma visits and deprivation by year of life with generalized linear mixed models.

**Results:**

OASIS asthma criteria were met for 21% of the 326,383 children. After adjustment for characteristics strongly associated with asthma, including male sex, prematurity, obesity, and atopic conditions other than asthma, children with high birth neighborhood deprivation were at increased risk of incident asthma (HR 1.11; 95% CI, 1.09–1.13). High deprivation in a given year of life was associated with increased odds of ongoing asthma during that year of life (OR 1.03; 95% CI, 1.02–1.05).

**Conclusions:**

Children living in high-deprivation neighborhoods are at increased risk of incident and ongoing asthma. This study suggests that neighborhood material deprivation may represent a helpful tool for evaluating the effects of disparities in health and social advantages on the likelihood of developing and continuing to need healthcare visits for ongoing childhood asthma.

## 1. Introduction

Parental income or income bracket is often used as a surrogate for child socioeconomic status (SES). Some cross-sectional and longitudinal studies have shown associations between parental income or SES and incident childhood asthma, but the associations have not been consistent [1–6]. Income alone may not take into account other home and family characteristics that represent the child's true SES; the need for a comprehensive measure of SES provided impetus for the development of the Material Deprivation Index.

Material deprivation, which takes absence of high school graduation, lone parent families, government transfers, unemployment, low income, and homes needing major repairs into account, as well as income, has been proposed as a more thorough measure of childhood SES and has been measured as a validated component of the Ontario Marginalization Index (ON-Marg) [7, 8]. Material deprivation allows examination of the effects of wide-ranging disparities in social well-being on chronic health conditions in children, addressing a gap in our ability to evaluate needs associated with lower SES among children.

Adults with high material deprivation have increased odds of asthma (OR 1.23; 95% CI, 1.17–1.28) [7]. Material deprivation has been associated with poor glycemic control in children with type I diabetes [9]. Stronger associations between childhood emergency department visits for asthma and air pollution have been demonstrated among children living in neighborhoods with high levels of deprivation [10]. To our knowledge, this index has not been studied in association with childhood asthma development or ongoing asthma not specific to emergency department visits. Neighborhood deprivation may represent a helpful tool for evaluating the effects of disparities in health and social advantages on the likelihood of developing and continuing to need healthcare visits for ongoing childhood asthma. We evaluated the associations between material deprivation and new-onset and ongoing childhood asthma among children living in the Greater Toronto Area (GTA).

## 2. Methods

### 2.1. Data Sources

We analyzed prospectively collected Ontario health administrative data regarding clinic visit records from the Ontario Health Insurance Plan (OHIP), emergency department records from the National Ambulatory Care Reporting System (NACRS), and hospitalization data from the Canadian Institute for Health Information-Discharge Abstract Database (CIHI-DAD), housed at the Institute for Clinical Evaluative Sciences (ICES). Outpatient visits to clinics and emergency departments and admissions to hospital for asthma are covered under provincial healthcare for all Ontario residents and are recorded in OHIP, NACRS, and CIHI-DAD for billing purposes. These data are complete for asthma visits of Ontario residents billed in the province of Ontario. We used the Registered Persons Database (RPDB) to identify children who were born between 1997 and 2003 and had lived in the Greater Toronto Area (GTA) at any time during their lives; these children's administrative data were followed from birth until March 31, 2012. Children born in 1997 had up to 15 years of data, and children born in 2003 had up to 8 years of data. This study was approved by Research Ethics Boards at the Hospital for Sick Children and the University of Toronto.

Material deprivation was developed by Matheson et al. to contribute to our understanding of inequalities in measures of health and social well-being and to allow standardization across various population groups and geographical areas. Material deprivation was generated using principal components factor analysis [7, 8] and has been validated by comparing 2001 and 2006 census tract measures and dissemination areas within the census tracts [7, 8]. The index was developed using mandatory self-reported data from 52,973 dissemination areas and 5,017 census tracts. Data were included in the index in aggregates by dissemination area, the smallest standard geographic area for which census data may be reported. To maintain confidentiality, data would not have been included for the smallest dissemination areas. The index was housed at ICES, which would not have reported aggregates of smaller than 6 families to prevent identification. Only aggregate data without individual identifiers were available to the investigators of this study.

The Material Deprivation Index comprises high school graduation, lone parent families, government transfers, unemployment, low income, and homes needing major repairs and ranges from a score of −2 (lowest deprivation) to +6 (highest deprivation). The index was reported in quintiles, with the lowest quintile being the least deprived and the highest quintile the most deprived. To align with other studies, material deprivation was dichotomized into the two highest quintiles versus the two lowest quintiles for the principal analysis.

The Ontario Asthma Surveillance Information System (OASIS) database, created in 2005, tracks individuals with reported healthcare administrative codes for asthma. Entry into the database requires two outpatient visits for asthma within two consecutive years or ever being hospitalized for asthma [11]. Children who meet the OASIS database entry criteria are followed longitudinally in the database, regardless of the presence or absence of future asthma visits. This system has been validated with good sensitivity and specificity against a clinical diagnosis of childhood asthma made by a physician [12] and by parental reporting of physician-diagnosed asthma in children [13]. Asthma encounters were defined by the OHIP or the primary diagnostic ICD-9/ICD-10 code in NACRS and CIHI-DAD (Supplemental Table 1). For children in the OASIS database, asthma visits recorded in the OHIP, NACRS, and CIHI databases were available for each year of life during which the child lived in Ontario. Incident asthma was defined as the timing of entry into the OASIS database. Ongoing asthma, distinguished from asthma that has been outgrown, was defined as at least one healthcare visit for asthma in a given year of the child's life.

We also considered covariables that had previously established association with incident asthma [1–3, 14, 15]; all potential covariables that were available in the administrative databases were included in the study. Data from the RPDB were used to determine sex, and data from the OHIP, NACRS, and CIHI databases were used to determine history of preterm birth, any diagnostic coding for obesity (weight or body mass index >95^th^ percentile), and atopic conditions other than asthma, including allergic rhinitis, eczema, and food allergy. Preterm birth, obesity, and atopic conditions were dichotomous variables, with codes for these conditions either present or absent for each child in the administrative datasets.

### 2.2. Statistical Analyses

All analyses were performed in SAS 9.3 (SAS Institute, Cary, NC). We conducted asthma-free survival analysis to compare the time to incident asthma for the highest 2 quintiles of home neighborhood deprivation versus the lowest 2 quintiles. Unadjusted and adjusted Cox proportional hazards models were used to determine the association between high deprivation at birth and age of incident childhood asthma diagnosis. The proportional hazards assumption was justified, no multicollinearity was observed between variables, and the Schoenfeld residuals showed good model fit.

Generalized linear mixed models (GLMM) with fixed intercepts were used to evaluate the associations between ongoing asthma (at least one asthma visit in each year of the child's life) and ongoing high deprivation in each year of the child's life. Results of fixed- and random-intercept GLMM were similar, and the random slope GLMM model had a negligible and nonsignificant slope for deprivation. GLMM is a subject-specific statistical method that accounts for the clustering or repeated measures of healthcare visits for asthma by year of life for each child. Generalized Estimating Equations (GEE) with an exchangeable covariance structure are population-average methods that were used to confirm the results of the GLMM analysis.

Each covariable was compared with deprivation and asthma in unadjusted models. In the adjusted models, all covariables were included as potential confounders and remained significant (*p* < 0.05) in the final adjusted model. Effect modification with neighborhood deprivation was evaluated for all confounders with *a priori* likelihood of interaction. Interaction terms were included in the model, and for variables showing evidence of statistically significant interaction, the models were re-run using indicator variables for each of the four categories of deprivation (0, 1) and each covariable (0, 1).

Histories of asthma, material deprivation, preterm delivery, obesity, and other atopic conditions were determined from Ontario administrative records and were complete for children who had lived in Ontario for all of their lives. Birth neighborhood deprivation data were complete for 94.3–97.3% of children depending on year of birth, and neighborhood deprivation data were complete for 94.3–96.2% of children in any given year. Children not born in an Ontario hospital (27.8%) may have been missing data regarding preterm birth, and children who did not live in Ontario throughout the study period may have been missing data regarding asthma, obesity, or other atopic conditions if they had been coded as having these diagnoses while living outside Ontario. To evaluate the possible effects of children moving into and out of the study area during the study period, we performed sensitivity analyses restricted to children born in an Ontario hospital and children living in the GTA during each year of their lives.

## 3. Results

There were 326,383 children with administrative healthcare records born from 1997–2003, inclusive, and living in Toronto. The OASIS criteria for incident asthma were met by 69,628 children (21.3%), with a median age of diagnosis of 2.5 years (interquartile range (IQR) 0.9–4.9); 14,050 children (4.3%) with a median age of diagnosis of 3.5 years (IQR 1.4–6.7) had ongoing asthma symptoms by ages 8–15 years, which would have excluded children with early-life wheezing that did not persist into later childhood.

In unadjusted proportional hazards models, high-deprivation birth neighborhood was associated with development of asthma (hazard ratio (HR) 1.14; 95% confidence interval (CI), 1.12–1.16) (Table 1). Male sex, preterm birth, obesity, and atopic conditions other than asthma were also associated with incident childhood asthma (Table 1). Obesity (HR 1.12; 95% CI, 1.11–1.13) and atopic conditions other than asthma (HR 1.09; 95% CI, 1.08–1.10) were also independently associated with high birth neighborhood deprivation. In proportional hazards models adjusted for sex, preterm delivery, obesity, and atopic conditions other than asthma, children with high birth neighborhood deprivation were at increased risk of incident asthma (HR 1.11; 95% CI, 1.09–1.13) (Table 1). The association between material deprivation and incident asthma persisted when the diagnosis of incident asthma was restricted to asthma development among children who continued to have visits for asthma at ages 8–15 years (HR 1.11; 95% CI, 1.06–1.15), excluding children with early-life wheezing that resolved by school age.

Children with high neighborhood deprivation in any year of life had increased odds of healthcare visits for asthma in that year (OR 1.03; 95% CI, 1.02–1.05) (Table 2). The analyses appeared to be robust, and the subject-specific and population-average results were similar. The covariables did not modify the associations between neighborhood deprivation and incident asthma or ongoing asthma.

Similar associations between high neighborhood deprivation and asthma were seen after restricting the analyses to children who were born in an Ontario hospital or children who had lived their whole lives in the GTA (Supplemental Table 2). Neighborhood material deprivation was strongly associated with neighborhood income quintile (odds ratio 75.6; 95% CI, 72.1–79.2), which was obtained from the RPDB and evaluated as a predictor of asthma development in separate models. Income quintile was associated with asthma development in unadjusted (HR 1.08; 95% CI, 1.06–1.11) and adjusted (HR 1.06; 95% CI, 1.03–1.08) models.

## 4. Discussion

Our results showed longitudinal associations between incident childhood asthma and neighborhood material deprivation at birth. Material deprivation in any year of life was also significantly associated with increased odds of visits for ongoing asthma in that year. The presence of an association between material deprivation and incident asthma among children who continued to have ongoing asthma until ages 8–15 years suggested that the association held true beyond transient early-childhood wheezing. The associations between childhood asthma development and material deprivation were robust and independent of the associations between childhood asthma development and male sex, prematurity, obesity, and other atopic conditions. Neighborhood material deprivation was associated with a higher hazard of childhood asthma development than neighborhood income quintile without overlapping confidence intervals and may have represented a more relevant measure for evaluations of incident asthma in children.

Studies of adults have shown material deprivation to be associated with asthma diagnosis [7, 8]. Stronger associations between childhood emergency department visits for asthma and air pollution have been demonstrated among children living in neighborhoods with high levels of deprivation [10]. Our study extends the findings of these previous studies by determining an association between material deprivation and new development of childhood asthma, rather than asthma exacerbations. We have also demonstrated an association with ongoing asthma, even among children who have not required emergency department visits.

Parental or household income has been commonly evaluated as a predictor of incident childhood asthma. A longitudinal Canadian study showed an increased hazard of incident childhood asthma at age 5 years among children with a low (HR 1.33; 95% CI, 0.98–1.78) and very low (HR 1.35; 95% CI, 1.01–1.82) SES index [1]. Neighborhood income has also been associated with incident childhood asthma in Toronto, Canada (HR 1.06; 95% CI, 1.03–1.09) [2]. However, a birth cohort of children in Manitoba, Canada [4], showed no association between family income and risk of incident asthma at age 7 years, after adjusting for maternal physician visits, hospitalizations, or prescription medications for depression or anxiety, suggesting that some of the variability in associations between childhood asthma and income may be due to other covariables considered in the models.

There is also evidence that the timing of income status may influence its association with incident childhood asthma. In a prospective Australian birth cohort, family income trajectories were modeled and physician-diagnosed asthma at age 14 years was associated with chronic low income (OR 2.21; 95% CI, 1.17–4.17) [5], while children of families with increasing and decreasing income did not have an increased odds of asthma at age 14 years. The variability of associations between income and incident childhood asthma, depending on the population and on the other covariables included, suggests that a more comprehensive measure of childhood well-being and SES should be used as a predictor in these models.

Mold and moisture damage is a frequently-studied characteristic related to home repair. Systematic reviews have generally shown associations between childhood asthma and home exposure to mold or moisture [16–19] although the results of individual cohort studies have been more variable [20–22]. In a nested case-control study of Canadian children, parent-reported visible mold exposure in pregnancy and childhood was not associated with asthma [20]. However, in a study of Taiwanese children without asthma, mold odor (OR 2.09; 95% CI, 1.30–3.37), parent-reported visible mold (OR 1.76; 95% CI, 1.18–2.62), and water damage (OR 2.80; 95% CI, 0.59–13.3) were associated with new-onset asthma [21]. In a Swedish nested case-control study, inspector-observed moldy odor along the skirting board was associated with asthma [22]. Home structural damage has been evaluated as a predictor of asthma control, and its improvement has been associated with improvement of asthma morbidity [23]. Although individual characteristics suggesting home disrepair have shown associations with asthma development, the associations have not been consistent across all studies. These findings underscore the possible utility of a more global measure of home repair being included in the evaluation of SES and its relationship with childhood asthma.

Other components of the deprivation index, including no parental high school graduation, lone parent families, government transfers, and unemployment, have been previously included as covariables in the longitudinal evaluation of associations with incident asthma in children. Single-parent families have shown an increased hazard of incident childhood asthma by age 5 years (HR 1.43; 95% CI, 1.17–1.76) [1]. Maternal receipt of social welfare has been associated with incident childhood asthma in a Canadian nested case-control study (OR 1.87; 95% CI, 1.43–2.44) [20]. Among elementary school children in southern California, Title I Funding supporting academic achievement in schools with >40% of children living in poverty has been associated with incident asthma (HR 1.68; 95% CI, 1.10–2.56) [24]. Low parental education (OR 2.62; 95% CI, 1.07–6.39) and unemployment (OR 2.38; 95% CI, 1.16–4.90) have been associated with maternal depressive symptoms, [25] which have in turn been associated with incident childhood asthma (OR 1.25; 95% CI, 1.01–1.55) [4]. Lower parental education has also been associated with asthma diagnosis in the first year of life (HR 1.32, 95% CI 1.18–1.47) [6].

Our study adds to the literature by demonstrating robust and novel associations between neighborhood deprivation and incident and ongoing childhood asthma in a large birth cohort of children. This study's strengths include its large sample size, unselected population of children, and the utilization of prospectively collected administrative data for the exposure, outcome, and covariables. We investigated a validated, comprehensive measure of material deprivation in children, which takes into account parental education, single parenthood, government transfers, unemployment, and homes in need of major repairs, as well as parental income. We also used a validated outcome measure with good sensitivity and specificity (91.4% and 82.9%, respectively) [12]. Sensitivity analyses showed that movement of children into and out of Ontario or the GTA did not substantially alter the associations between material deprivation and childhood asthma.

Neighborhood-level material deprivation data may not apply to all individuals within a neighborhood. However, individual measures of factors making up the deprivation index also have limitations. Studies of asthma symptoms have suggested that individual and neighborhood measures of poverty may be independent of each other in some populations [26]. Longitudinal evaluation of neighborhood has been shown to be critical for accurate determination of associations with health outcomes [27]. Individual-level family income and home repair data also pose potential problems, including higher rates of missing data if people prefer not to report their income or state of home disrepair. Parental or observer reports of mold and moisture damage are difficult to standardize among different studies, which may also detract from the usefulness of individual-level data regarding home disrepair.

This study was conducted in a large, cosmopolitan, Canadian, urban center and may not be generalizable to smaller urban centers or rural communities. We did not have information regarding family history of asthma and personal exposures such as second-hand smoke inside the home or at other locations. Individual-level cohort studies will also be needed to re-evaluate the associations of these covariables with material deprivation.

## 5. Conclusions

Our results show longitudinal associations between home neighborhood material deprivation and incident childhood asthma and ongoing asthma in each year of the child's life. This study demonstrates the utility of a validated measure of material deprivation in studies of childhood asthma. Neighborhood material deprivation may represent a helpful tool for evaluating the effects of disparities in health and social advantages on the likelihood of developing and continuing to need healthcare visits for ongoing childhood asthma.

## Figures and Tables

**Table 1:** Associations between childhood asthma development and birth neighborhood deprivation and covariables.

Predictor	*N* (%)^b^	Unadjusted HR (95% CI)^c^ of incident asthma	Adjusted HR (95% CI)^c^ of incident asthma
High deprivation	125 664 (38.5)	1.14 (1.12, 1.16)	1.11 (1.09, 1.13)
Male sex	167 560 (51.3)	1.37 (1.35, 1.39)	1.43 (1.41, 1.46)
Preterm birth	22 639 (6.9)	1.56 (1.53, 1.59)	1.58 (1.54, 1.62)
Obesity	18 071 (5.5)	1.55 (1.52, 1.59)	1.46 (1.42, 1.51)
Other atopic conditions^a^	222 667 (68.2)	2.79 (2.73, 2.84)	2.96 (2.89, 3.03)

^a^Atopic conditions other than asthma (allergic rhinitis, food allergy, and atopic dermatitis). ^b^Percentages account for missing data. ^c^Hazard ratio (95% confidence interval). Results of multivariable Cox proportional hazards models.

**Table 2:** Associations between asthma visits and neighborhood deprivation and covariables in each year of life among children living in the Greater Toronto Area.

Predictor	OR (95% CI)^b^ of asthma visits by year using fixed-intercept GLMM^c^	OR (95% CI)^b^ of asthma visits by year using GEE^d^
High deprivation	1.03 (1.02, 1.05)	1.03 (1.02, 1.05)
Male sex	1.36 (1.34, 1.37)	1.37 (1.36, 1.39)
Preterm birth	1.47 (1.44, 1.51)	1.52 (1.48, 1.56)
Obesity	1.48 (1.45, 1.51)	1.47 (1.44, 1.51)
Other atopic conditions^a^	2.82 (2.77, 2.87)	2.79 (2.74, 2.85)

^a^Atopic conditions other than asthma (allergic rhinitis, food allergy, and atopic dermatitis). ^b^Adjusted odds ratio of asthma (95% confidence interval), also adjusted for year of birth. Results of ^c^generalized linear mixed model (subject-specific model) and ^d^Generalized Estimating Equations (population-average model).

## Data Availability

Data for this project were accessed and generated within the Institute for Clinical Evaluative Sciences (ICES) Data Repository. Approval from ICES is necessary to access the data.

## Abbreviations

CI: Confidence interval
CIHI-DAD: Canadian Institute for Health Information-Discharge Abstract Database
GEE: Generalized Estimating Equations
GLMM: Generalized linear mixed models
GTA: Greater Toronto Area
HR: Hazard ratio
ICES: Institute for Clinical Evaluative Sciences
IQR: Interquartile range
NACRS: National Ambulatory Care Reporting System
OASIS: Ontario Asthma Surveillance Information System
OHIP: Ontario Health Insurance Plan
ON-Marg: Ontario Marginalization Index
OR: Odds ratio
RPDB: Registered Persons Database
SES: Socioeconomic status.

## Supplementary Materials

Supplementary Materials Supplemental Table 1: OHIP, ICD-9, and ICD-10 diagnostic codes. Supplemental Table 2: Associations between incident asthma and birth neighborhood deprivation among subgroups of children living in the Greater Toronto Area (GTA).
