# Supplementary material for: Neighborhood Material Deprivation Is Associated with Childhood Asthma Development: Analysis of Prospective Administrative Data
Source: Can Respir J. 2019 May 19;2019:6808206. doi: 10.1155/2019/6808206 (PMC6545816; doi:10.1155/2019/6808206)
Supplement: Supplementary Materials — Supplemental Table 1: OHIP, ICD-9, and ICD-10 diagnostic codes. Supplemental Table 2: Associations between incident asthma and birth neighborhood deprivation among subgroups of children living in the Greater Toronto Area (GTA). [file 6808206.f1.pdf]

Supplemental Table 1. OHIP, ICD-9 and ICD-10 diagnostic codes

Condition	OHIP	ICD-9	ICD-10
Asthma	493	493	J45
Preterm delivery	765	765.00 to 765.19	P070, P071, P072, P073
Obesity	278	278.00	E660, E668, E669
Other atopic conditions			
Allergic rhinitis	477	477.00, 477.08, 477.09	J301, J302, J303, J304
Atopic dermatitis	691	691.80	L208, L209
Food allergy	995	995.00	T7800 to T7809

Supplemental Table 2. Associations between incident asthma and birth neighborhood deprivation among subgroups of children living in the Greater Toronto Area (GTA)

	Children born in an Ontario hospital Hazard Ratio (95% CI)[a]	Children living continuously in the GTA Hazard Ratio (95% CI)[a]
High deprivation	1.18 (1.15, 1.20)	1.11 (1.08, 1.13)
Male sex	1.44 (1.42, 1.47)	1.45 (1.42, 1.49)
Prematurity	1.32 (1.29, 1.36)	1.59 (1.54, 1.65)
Obesity	1.35 (1.31, 1.39)	1.49 (1.43, 1.54)
Atopic conditions	1.98 (1.92, 2.03)	2.87 (2.79, 2.96)

[a] Adjusted hazard ratio of incident asthma (95% confidence interval), also adjusted for year of birth. Results of multivariable Cox proportional hazards models
